# Crop climate suitability mapping on the cloud: a geovisualization application for sustainable agriculture

**DOI:** 10.1038/s41598-020-72384-x

**Published:** 2020-09-23

**Authors:** Brad G. Peter, Joseph P. Messina, Zihan Lin, Sieglinde S. Snapp

**Affiliations:** 1 Department of Geography, University of Alabama, Tuscaloosa, AL 35487 USA; 2 Department of Geography, Environment, and Spatial Sciences, Michigan State University, East Lansing, MI 48824 USA; 3 Department of Plant, Soil and Microbial Sciences, Michigan State University, East Lansing, MI 48824 USA

**Keywords:** Agroecology, Biogeography, Phenology, Climate-change adaptation, Sustainability, Information technology

## Abstract

Climate change, food security, and environmental sustainability are pressing issues faced by today’s global population. As production demands increase and climate threatens crop productivity, agricultural research develops innovative technologies to meet these challenges. Strategies include biodiverse cropping arrangements, new crop introductions, and genetic modification of crop varieties that are resilient to climatic and environmental stressors. Geography in particular is equipped to address a critical question in this pursuit—when and where can crop system innovations be introduced? This manuscript presents a case study of the geographic scaling potential utilizing common bean, delivers an open access Google Earth Engine geovisualization application for mapping the fundamental climate niche of any crop, and discusses food security and legume biodiversity in Sub-Saharan Africa. The application is temporally agile, allowing variable growing season selections and the production of ‘living maps’ that are continually producible as new data become available. This is an essential communication tool for the future, as practitioners can evaluate the potential geographic range for newly-developed, experimental, and underrepresented crop varieties for facilitating sustainable and innovative agroecological solutions.

## Introduction

A grand challenge of today is achieving food security in an era confronting global environmental and societal change^1,2^. In recent decades, food shortage has remained a chronic issue in many Sub-Saharan African countries, in part due to insufficient production resulting from climatic stressors, environmental degradation, and population growth^3,4^. Droughts, unpredictable rainfall patterns, and severe floods have led to substantial fluctuations in agricultural output^5–7^, notably where biophysical conditions are suboptimal or where social factors limit use of inputs to enhance productivity^8^. As Lobell et al.^9^ observed across 1980–2008, declines in global production of maize and wheat can be attributed to shifting climate trends, potentially incurring substantial economic losses and disrupting farmer livelihoods^3^. Unusually high temperatures have negatively impacted yields^10,11^, facilitated food spoilage^12^, and altered crop geographic niche and plant biodiversity^13^. Moreover, agricultural systems are susceptible to compounding impacts from climate change, such as the change in geographic distributions of pests and diseases that damage crops and impact yields^14^.

Agrotechnological advancements are occurring at a rapid rate to address these challenges and elucidating the spatial organization of crop suitability is critical for informing where solutions can be deployed that improve crop system efficacy^1^. Knowledge of where to implement, with reasonable probability of success, is at the foundation of sustainable strategies for intensifying food production^15^. In the geospatial sciences, there is an increasing abundance of climate and biophysical data, improved frequency of satellite image collection and delivery, and myriad computational tools capable of processing large volumes of geospatial data^16^. The new geographic information science platforms and biogeographic crop suitability maps possible today offer considerable value for enhancing the impact of agricultural improvement across the world^17^. As climate patterns shift and new cropping system strategies emerge, it is vital to ‘widen the approach’ by generating continuous geospatial knowledge and products^18^. Given the concerns regarding future climate change impacts on global food security and the need to sustainably produce more food^19^, where can alternative cropping systems be introduced?

One challenge associated with scaling agricultural innovations is that biophysical constraints (e.g., climate and soil properties) are often neglected or misrepresented when targeting areas for technology implementation; in some cases, regional aggregations or the use of distant weather stations will inaccurately characterize areas that experience unique local climates^20^. There are many reasons why crop introduction might fail to achieve long-term adoption or result in unsuccessful harvests, such as socioeconomics, governmental policy, demographics, and infrastructure^21–23^. However, a climatic reason for crop failure may be predictable and avoidable based on geospatial climate data alone. Biophysical suitability, knowledge of plant fundamental niche, and understanding of regional food cultures are necessary first conditions for adoption and upkeep of new or updated crop varieties^24^. Fundamental niche is characterized by plant physiological responses to ecosystem constraints (e.g., temperature, precipitation, and soil status), whereas realized niche addresses species distribution based on observation^25^.

Satellite image models are prominent tools for supplying spatially continuous weather and climate data, yet there is a lack of accessible and updatable geospatial products to support targeting of crop system innovations. Further, there are steep barriers to broader utilization of geospatial data such as costly software and expert knowledge. This makes it problematic for transdisciplinary research bridging geography and agronomy to access geovisualizations and spatial suitability products. At present, the most notable crop suitability maps are the Global Agro-Ecological Zones (GAEZ) products distributed by the Food and Agriculture Organization of the United Nations (FAO)^26^. While these products are immensely valuable, they are not produced at regular or frequent time intervals, and there are uncertainty issues embedded in the products that utilize future climate predictions^27^. As global changes continue to occur, geospatial analytics and map outputs will need to be generated with greater frequency to produce dynamic, ‘living maps’^28^.

### Paradigm shift: crop niche mapping on the cloud

Remote sensing-based characterizations of crop suitability are regularly harnessed to visualize the spatial distribution of crop suitability and production potential. Conventionally, such mapping efforts occur at singular time intervals, for specific crops, and use classifications driven by complex algorithms. Even the most widely-used land and crop suitability maps are subject to diminishing relevance over time, particularly in the face of rapid global change. Existing tools for crop suitability mapping consist of tabulating where crops are currently produced or modeling where crops may be able to grow^29,30^. The simulation approach predicts crop growth and response to variable climate, soil quality, and management strategies using complex plant physiology algorithms. Each crop suitability model is developed for a different purpose; for example, AquaCrop was designed to explore, analyze, and estimate the impact of water supply on crop viability^31^ and DIVA-GIS was designed as an open access tool to map the geographic distribution of crop species and facilitate climate data extraction^32^. Other complex models, such as DSSAT (Decision Support System for Agrotechnology Transfer)^33^ and APSIM (Agricultural Production Systems sIMulator)^34^, simulate crop yields and farm soil status based on parameters set by the user, embedded databases and functions (e.g., crop rotations and arrangements), and meteorological measurements (e.g., solar radiation, temperature, and rainfall). These models are typically desktop-based and operate client-side on a computer or lab workstation. Model simulations are important research tools; however, they require specialized training and expert knowledge to produce relevant results.

Web-based GIS (geographic information science/systems) has the potential to transform the crop niche mapping paradigm, bringing series of static maps to a dynamic form complete with interactive geovisualizations and temporal continuity^35,36^. Acquisition and preprocessing of large-scale time-series satellite imagery is often time-consuming and requires arduous data management, expensive computational hardware, software licenses, and storage structures capable of processing and moving large volumes of data. Change detection over long time scales and vast spatial extents can require weeks of data organization and petabytes of storage space. As technological advancements refine the spatial and temporal resolution of global imagery, challenges associated with data storage and processing compound. Recently, however, cloud-based alternatives are offering increased efficiency for large-scale geospatial computing. In December of 2010, Google launched Google Earth Engine (GEE) at the International Climate Change Conference in Cancún, Mexico^37^. GEE is a web-based high-computational platform that facilitates unprecedented planetary-scale satellite imagery access and geospatial analytics^38^. Server-side data processing and imagery archival removes many of the data storage and geoprocessing hardware demands required for remote sensing and geographic information science/systems (GIS). The emergence of GEE supports improved data accessibility, data processing efficiency, and scalability of geographic solutions^38,39^.

### Case study: legume biodiversity in Africa

At the continental scale, Africa contains many agroecological zones that can support a wide variety of crops^40^; however, many ecosystem service-offering crops are underrepresented by global suitability modeling studies^41,42^ and underutilized in existing agricultural systems^43^. Promotion of biodiverse cropping systems that offer multiple ecosystem services, such as those that leverage nitrogen fixation from legume integration (e.g., pigeonpea and maize intercropping), has gained traction in recent years^44–46^, due in part to sustainability concerns associated with the continuous production of maize leading to widespread soil nutrient and organic matter depletion^47^. However, Foyer et al.^48^ argue that human nutrition and agricultural sustainability have been negatively impacted by an overall lack of emphasis on legume integration in existing crop systems.

A prominent area of research for plant geneticists and crop breeders is the development of crop varieties that are resilient to abiotic stressors such as marginal temperature, rainfall, and soil nutrient deficiencies^49^. Drought-tolerant varieties of pigeonpea, for example, contain traits that can be exploited to remain yield-stable during water scarce periods^50^, and short-duration plant varieties can mature and produce harvestable grain during short unimodal rains^51^. Cowpea is regarded as a stress-tolerant crop, with cultivars demonstrating resilience to dry and hot environments, such as those in the Sahel and Sub-Saharan Africa, as well as resistance to pests and diseases by way of early maturation^52^. Like cowpea, cultivars of common bean (*Phaseolus vulgaris* L.) contain traits that enable its production in marginal environments where biotic and abiotic stressors, such as drought, soil nitrogen deficiency, pest infestations, and disease, can inhibit crop productivity^53,54^. In addition, common bean is efficient in its biological fixation of nitrogen, garnering appeal in regions experiencing soil nutrient degradation^53^. Moreover, fast-maturing varieties are a high-protein source of nutrition during food scarce periods and mounting evidence shows high uptake of common bean by smallholder farmers since the 2000s^55^. Problematically, however, projections of hotter temperatures threaten the geographic niche of common bean production across Sub-Saharan Africa^56^, prompting rapid genetic research for temperature resilient breeds and highlighting a need for dynamic crop suitability map products.

Despite the deep history of legume production in Sub-Saharan Africa, the limited area under pulse production does not coincide with the biophysically suitable niche for many legume species^55^, due in part to colonial displacement of indigenous legume crops^57^. This increasingly narrow range of crop species grown is mirrored over much of the globe, which likely reduces farm resilience and food security during extreme climatic events, as plant biodiversity is an important buffering mechanism^6,58^. Consequently, renewed attention has been given to legume diversification on smallholder farms in Sub-Saharan Africa, not only for soil rehabilitation and farm resilience, but also as complementary nutrition in household consumption^59–61^. Smallholder farmers depend on consistent production to sustain their livelihoods, both for household economics and subsistence, and it is necessary that crop varieties selected are appropriate for specific geographical limitations, input availability, and regional food culture^24,62^. Considering the generational knowledge and ecological history of legume production in Sub-Saharan Africa, and the persistence of indigenous pulses such as cowpea (*Vigna unguiculate* L. Walp.), re-integration of many legume varieties poses promising long-term adoption outcomes in both the environmental and social contexts^63,64^.

### The ‘when’ and ‘where’ of crop suitability

The research and application presented here was conceptualized after extensive interdisciplinary collaboration among geographers, plant and soil scientists, crop system modelers, and climate scientists. The paper focuses on an important question—When and where is crop niche?—and explores specifically (a) the geographic extent of climate suitability for common bean (*Phaseolus vulgaris* L.) across Southern and Southeastern Africa, as well as (b) a hypothetical scenario illustrating the geographic area that would shift to a suitable status if a common bean variety was bred to withstand temperatures of 2 degrees Celsius higher. This case study is concordant with ongoing research recognizing the utility of bean varieties with improved heat tolerance^65^. A similar approach is documented in recent publications that explored legume crop options, suitability for development, and the current extent of legume production^44,55^. As agronomic and genetic development expand the geographic niche of certain crops, more efforts of this kind will be required for the future of food security.

This paper moves beyond static mapping into a dynamic and continuously updatable form, and the geocommunications supplied herein contribute to the global body of knowledge geared toward narrowing the research-implementation gap^66^. To address stated geocommunication and accessibility goals, a crop climate-suitability application was developed using the GEE platform that spans space, time, and crops. Suitability visualization outputs are based on the fundamental climate niche and phenology of the crop selected; the maps can also be queried for climate, soil quality, and terrain characteristics at any location. The mapping interface is equipped with a graphical user display, allowing crop phenology selections, modifiable time period observations, and multiple geographic area delineations. Leveraging cloud-based technology can improve the accessibility of global climate information for crop niche mapping, granting more geographic decision power to agronomists, farmers, conservationist stakeholders, and government policymakers to effectively scale agricultural improvement and ultimately illuminate what crops can grow where and when. Compared to existing crop suitability models and maps, the crop niche application presented here is cloud-based, quasi-global, geographically and temporally agile, and is accessible to a broad range of users. Geospatial datasets are stored server-side, removing the burden on users to acquire, filter, and organize data for models with specific formatting requirements and steep barriers to entry.

## Results

The geovisualization application was developed as an accessible tool for delineating crop climate suitability and empowering practitioners and academics across disciplines to engage with geographic information. The results first describe the application user interface, inputs, and outputs, and then demonstrate a use case by assessing the fundamental climate niche in Africa for common bean (i.e., the potential geographic range based on temperature and rainfall suitability). Maps are presented that elucidate how crop suitability can be spatially expanded through breeding a common bean variety that withstands hotter temperatures. Also highlighted is the temporal flexibility of the model application through (1) a panel comparison of common bean niche in Tanzania across moving 4-month interval growing seasons and (2) a juxtaposition of two individual years to show crop suitability during a year characterized by extreme climate (i.e., low rainfall and high temperatures) as compared to a year more indicative of a good production season (measured via NDVI).

The process for map production involved temperature, precipitation, and NDVI data acquisition/aggregation for the date range and season range selections (aggregated by season first, then aggregated across selected years) and geographic delineation chosen, then suitability characterization based on the temperature and rainfall crop phenology parameters selected by the user. Data products, suitability characterization methods, and crop phenology parameters are detailed in the methods section.

### Crop niche web application interface

The application is available online at https://cropniche.cartoscience.com or https://cartoscience.users.earthengine.app/view/crop-niche. The crop climate-suitability application has six user input fields (Table 1). First, the user may select a boundary of interest, which includes three options—country selection, user-defined region, or quasi-global. The user-defined region is a rectangular area using the centroid coordinates entered and a buffer distance; this selection may be preferred if the user is interested only in a localized area and needs to minimize processing time. The quasi-global boundary computes all available data, but is restricted by the extent of the CHIRPS precipitation data product, which covers the tropical range extending from 50°N to 50°S^67^. If the user-defined region or the quasi-global options are not checked, the application will default to the country selected from the dropdown widget. Note that larger extents will require more processing time; if prompted, wait for the browser to respond while data layers are loaded.

**Table 1:** Input options, output products, and features of the geocommunication application. The application is available online at https://cropniche.cartoscience.com or https://cartoscience.users.earthengine.app/view/crop-niche.

User input	Geospatial data layers	Queried information
**Data bounds** (1) Country, user-defined region (decimal degrees centroid and buffer distance), or quasi-global (tropics, 50°N–50°S) **Temporal aggregations** (2) Range of years (yyyy) and (3) crop growth season duration in months/days (MM-dd) **Crop phenological requirements** (4) FAO ECOCROP parameters or (5) user-defined rainfall ranges during growth season and (6) temperature ranges during growth season	**Suitability characterization** (1) Temperature, rainfall, combined, and combined masked to agriculture **Boolean suitability** (2) Combined, temperature, and rainfall **Climate and land data** (3) Average seasonal temperature, average seasonal rainfall amount, average seasonal NDVI, and agricultural land-use	**Soil and terrain** Average seasonal rainfall, average temperature, average NDVI, organic carbon, bulk density, pH in H_2_O, soil water content at 33 kPa, sand/silt/clay fractions, biome and taxonomy classifications, elevation, and slope **Time-series charts** Both pixel and regional temperature, rainfall, and NDVI trends **Data extraction** Time-series seasonal temperature, rainfall, and NDVI at the pixel and regional levels

The second and third input fields are the temporal aggregations. This section differentiates the application from many other crop niche mapping methods in that it can accommodate seasons that wrap over the new year. In the case of Malawi, the rainy season spans from November to March^68,69^; if data from a single year are aggregated, the results would be based on two different growing periods. Averaging temperatures across multiple growing seasons and multiple years may introduce marginal error; however, for rainfall amounts the problems may be substantially greater given that there are episodic droughts and floods during some seasons. This is where practitioners can use expert knowledge to customize season ranges to account for short- and long-duration crop varieties that are underrepresented by other crop mapping models.

At the time of manuscript construction, the available data date range is from 2000-02-18 to 2019-11-26 (available data date range at the time of use is posted in the application interface). One of the novelties of using this platform is that the data availability end date continues to update as new data are collected and entered into GEE; new maps can be created at any time. If the season (selected in input 3) wraps over the new year, data will be accessed from the year following the end year selected here (e.g., if 2018 is the last year selected for a November to April season, 2018-11-01 to 2019-04-30 will be used). Note that longer time periods will require more processing time.

Input 4 allows the user to select a crop from a dropdown box that utilizes temperature and precipitation ranges from the FAO ECOCROP database^70^. Available crops included here are barley, cassava, chickpea, common bean, cowpea, green peas, groundnut, lentils, maize, millet, pigeonpea, potato, quinoa, rice paddy, sorghum, soybean, sugarcane, sunflower, sweet potato, tobacco, and wheat. Parameters used from this database are posted alongside the outputs after processing is complete. If the checkbox in input 4 is selected, no climate parameters are required—input options 5 and 6 can be ignored and maps will be produced using the FAO ECOCROP database parameters. Optionally, the user may elect to enter custom climate parameters for any crop variety in inputs 5 and 6; in this case, the dropdown in input 4 can be ignored and the checkbox should remain unchecked. Inputs 5 and 6 are where practitioners can use expert knowledge to customize temperature and rainfall ranges for crop varieties that are underrepresented by other map models.

There are currently ten data layers of three types loaded to the display after user input options are selected (Table 1). The first type is suitability characterization using the parsing approach discussed in the methods section, which includes optimal, suitable, marginal, unsuitable, and pessimal categories^44^; available data layers for display in this category include (i) temperature suitability, rainfall suitability, combined suitability (the minimum of the temperature and rainfall suitability layers), and combined suitability masked to the agricultural land-use extent. The second data layer type is the boolean suitability category; these data layers are labeled as suitable or not suitable and have not undergone any additional characterization and are based solely on the input temperature and precipitation ranges. The third type consists of summary climate and land data. Available data layers include average seasonal temperature, average seasonal rainfall amount, average seasonal NDVI, and agricultural land-use extent.

Climate, soil, and terrain information can also be queried per pixel. The user can click the map to retrieve pixel scale average seasonal rainfall, average seasonal temperature, average seasonal NDVI, organic carbon, bulk density, pH in H_2_O, soil water content at 33 kPa, sand/silt/clay fractions (averaged across 0–30 cm depths), biome and taxonomy classifications, elevation, and slope. Time-series charts are also produced at the regional scale and per pixel. Each data point in the charts is the average across the selected season (x-axis labels are the season start date) and can be expanded to export as a CSV or another file type. The application also returns the user-selected parameters and a list of each season used for data aggregation so that results can be interpreted accurately. To enhance usability, a simplified version of the model for the GEE JavaScript interface is accessible via Harvard Dataverse (https://doi.org/10.7910/DVN/UFC6B5); this version performs the same computations, but gives experienced GEE users more control over the process and allows for map output exporting^71^.

### Fundamental climate niche of common bean

**Figure 1:**
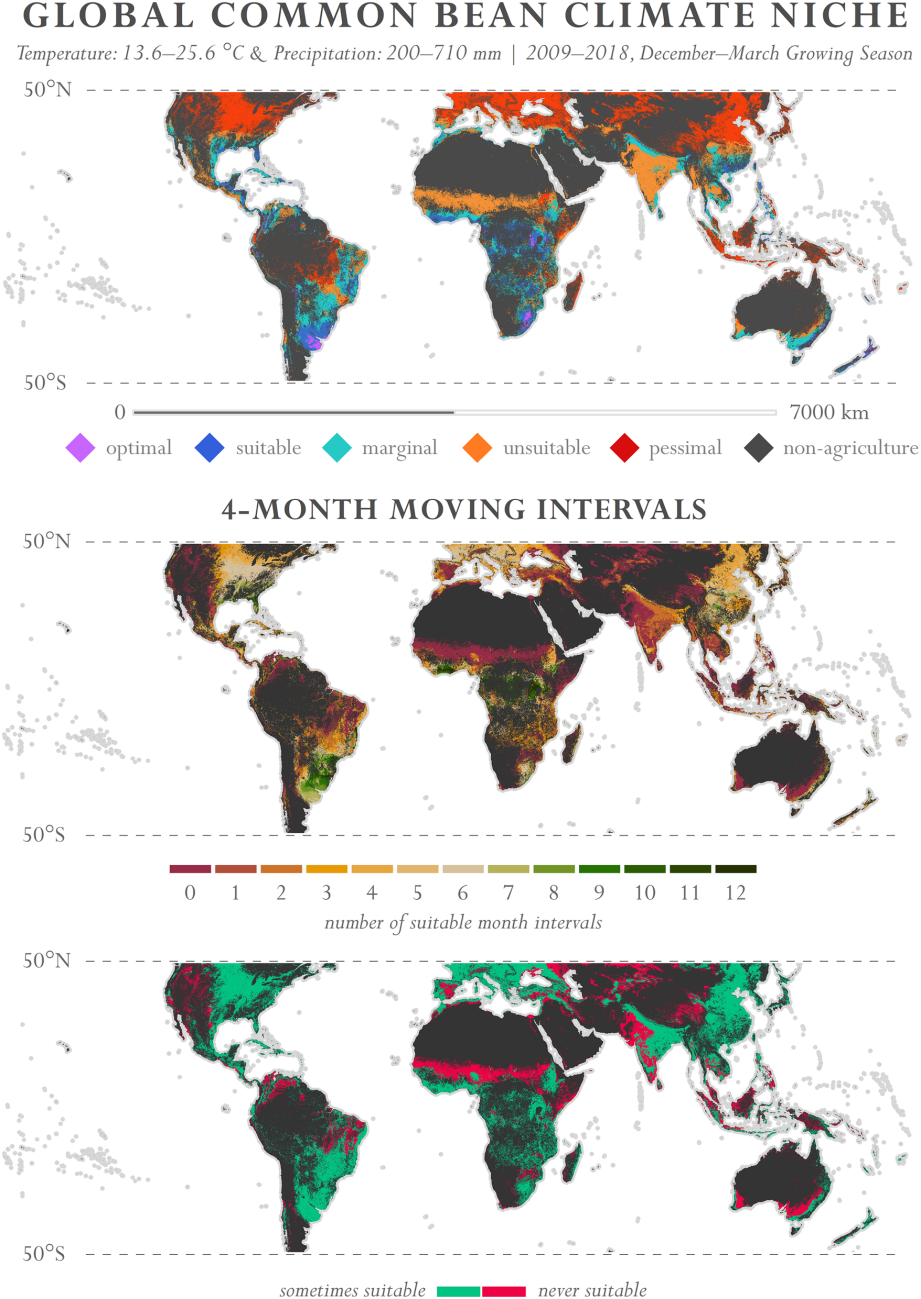
Quasi-global map of common bean suitability. Temporal range: 2009–2018; precipitation range: 200–710 mm; temperature range: 13.6–25.6 °C. *Top*—Single 4-month interval (December–March). *Middle*—Number of 4-month intervals that exhibit common bean suitability. The month intervals of October–January, November–February, and December–March use 2008–2017, whereas the remaining month ranges use 2009–2018. This decision was made so that the moving interval windows would be sequential. *Bottom*—Areas that are either never suitable or sometimes suitable (at least 1 of the 4-month intervals) for common bean production. Imagery processed using GEE^38^ and figure prepared using ArcGIS^96^. Data sources: CHIRPS precipitation^67^ and NASA MODIS LST^85^.

**Figure 2:**
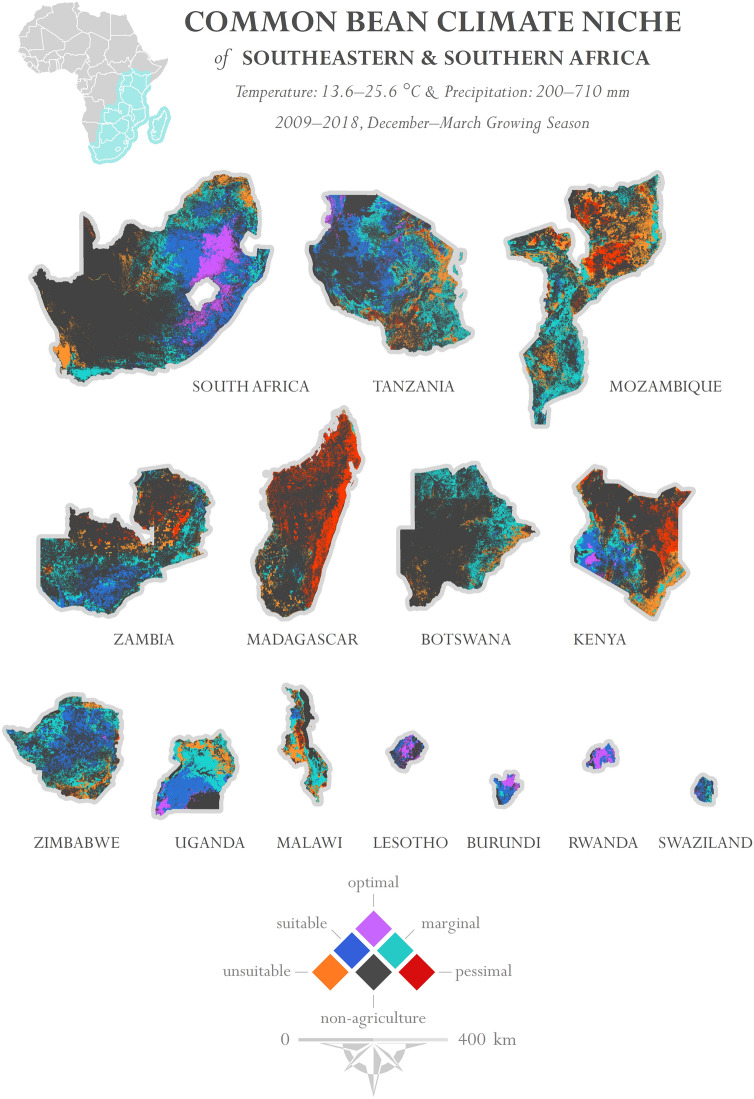
Map of common bean suitability in Southeastern and Southern Africa. Growing season months: December–March; temporal range: 2009–2018; precipitation range: 200–710 mm; temperature range: 13.6–25.6 °C. Imagery processed using GEE^38^ and figure prepared using ArcGIS^96^. Data sources: CHIRPS precipitation^67^ and NASA MODIS LST^85^.

We explore here the potential for crop varieties to scale across continents. A case study is presented on the geographic potential for common bean to be grown under current crop species phenological requirements. The suitable baseline parameters for common bean used here are from Beebe et al.^72^, including the temperature range of 13.6–25.6 °C and 200–710 mm of rainfall. A quasi-global map of common bean suitability is presented in Fig. 1. Suitability maps were also produced for select countries in Sub-Saharan Africa using the same parameterization—South Africa, Tanzania, Mozambique, Zambia, Madagascar, Botswana, Kenya, Zimbabwe, Uganda, Malawi, Lesotho, Burundi, Rwanda, and Swaziland (Fig. 2).

It is important to note that these results can be adjusted and updated in real time, based on the growing season selected and the number of years used to aggregate data and typify the climate. An underlying effect to acknowledge here is the modifiable temporal unit problem (MTUP), a term used to acknowledge data aggregation differences resulting from variations in selected temporal range, temporal resolution, and/or the particular period or point in time observed^73^. To address MTUP, Fig. 1 also depicts the number of 4-month intervals that exhibit common bean suitability (i.e., January–April, February–May, March–June, and so on). Here temperature and precipitation are aggregated by the month range selected, then averaged across the complete temporal range before parsing into suitability categories. By evaluating each interval, areas that are either never suitable or sometimes suitable for common bean production can be determined (Fig. 1). This method can be used as a universal definition of suitability; however, it is still necessary to test specific month ranges to evaluate when crop production can occur. Similarly, it is critical to evaluate crop suitability during individual years to better understand whether or not a plant can withstand temperatures and rainfall amounts during extreme years (e.g., droughts and unusually high temperatures). Considering that climate varies by region, plant requirements vary by crop, and the optimal scale of analysis can vary depending on the research goal, the application presented was designed with temporal flexibility so that expert knowledge can be used to define time ranges for observation.

**Figure 3:**
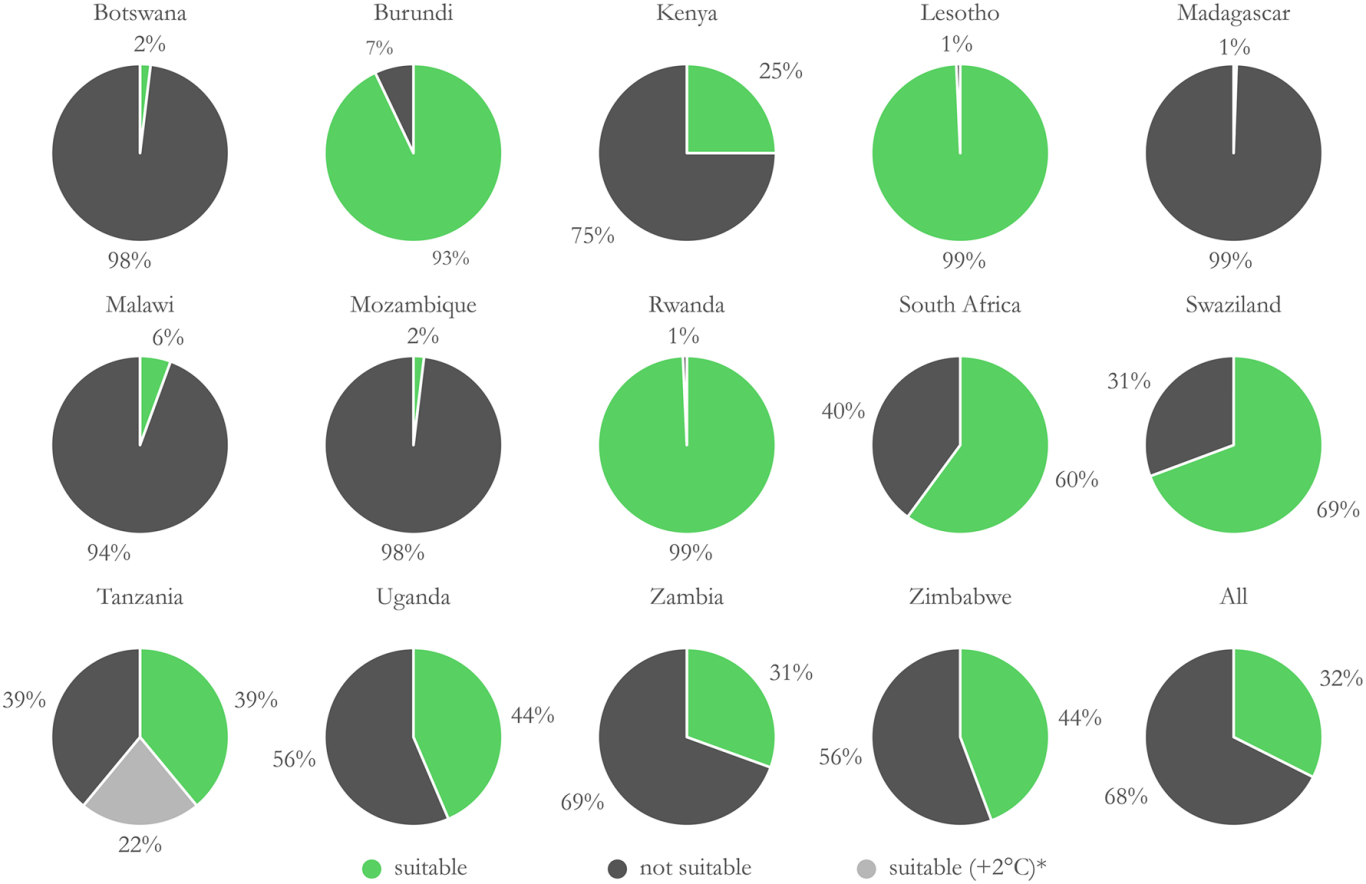
Suitable and not suitable agricultural land area proportions by Southeastern African countries. Growing season months: December–March; temporal range: 2009–2018; precipitation range: 200–710 mm; temperature range: 13.6–25.6 °C. Refer to Fig. 2. *Tanzania includes the hypothetical temperature scenario. Refer to Fig. 4. Under the baseline suitability scenario, 61% of Tanzanian agricultural land is not suitable for common bean production according to the parameters selected.

There is a substantial difference in common bean suitability across the Southern and Southeastern countries of Africa during the December to March production season. As stated above, MTUP plays a major role in these results. During this particular season, Lesotho, Rwanda, and Burundi exhibit pervasive common bean suitability, with 93–99% of agricultural land climatically fit for its cultivation. Larger countries such as South Africa and Tanzania show partial suitability; however other geographic areas may be suitable during other times of the year (Fig. 3).

Malawi and Mozambique occupy a similar agroclimatological zone and both countries show overall low suitability during these months. Given the latitudinal rainfall patterns in Sub-Saharan Africa, another range of months is likely more appropriate for Malawi and Mozambique. Considering that common bean is grown widely in Malawi, we tested suitability during a January to April production season and found that the suitable niche expanded from 6 to 59%. This is also observed in Tanzania, where the suitable area expands from 50.4% during the January–April season to 66.8% during the February–May Season (Fig. 5, Table 3), providing further evidence against a one-size-fits-all approach to crop suitability assessments. This demonstrates that MTUP is critically important to consider when testing for crop suitability.

**Figure 4:**
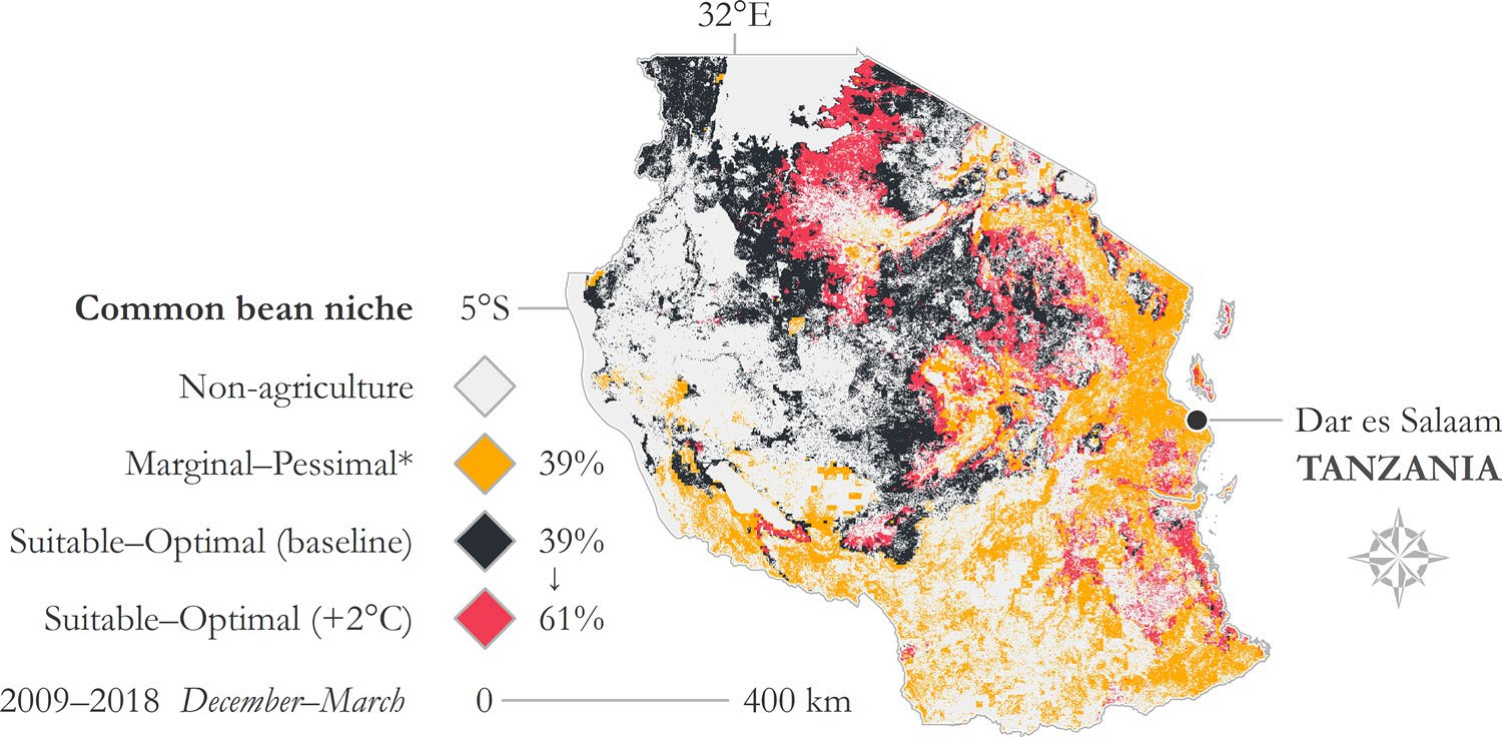
Map illustrating hypothetical suitable area gains from a common bean cultivar that can withstand 2 °C higher in Tanzania. Growing season months: December–March; temporal range: 2009–2018; precipitation range: 200–710 mm; baseline temperature range: 13.6–25.6 °C; temperature range for the hypothetical scenario: 13.6–27.6 °C. *Marginal–Pessimal area proportion tabulated in the figure is for the 13.6–27.6 °C temperature scenario. Imagery processed using GEE^38^ and figure prepared using ArcGIS^96^. Data sources: CHIRPS precipitation^67^ and NASA MODIS LST^85^.

Suitability was tested in Tanzania for two scenarios—(1) current temperature and precipitation requirements for common bean (referred to herein as the baseline) and (2) a simulated heat tolerant variety with the maximum temperature raised by 2 °C. The growing season, precipitation range, and minimum temperature were held constant. Under the current baseline requirements, 39% of agricultural land in Tanzania meets temperature and rainfall requirements for common bean production; however, if a common bean variety could withstand temperatures of 2 °C higher, 22% more agricultural land could be accessed for its cultivation (Fig. 4). In this scenario 61% of Tanzania is suitable for common bean production and 39% is not suitable.

**Figure 5:**
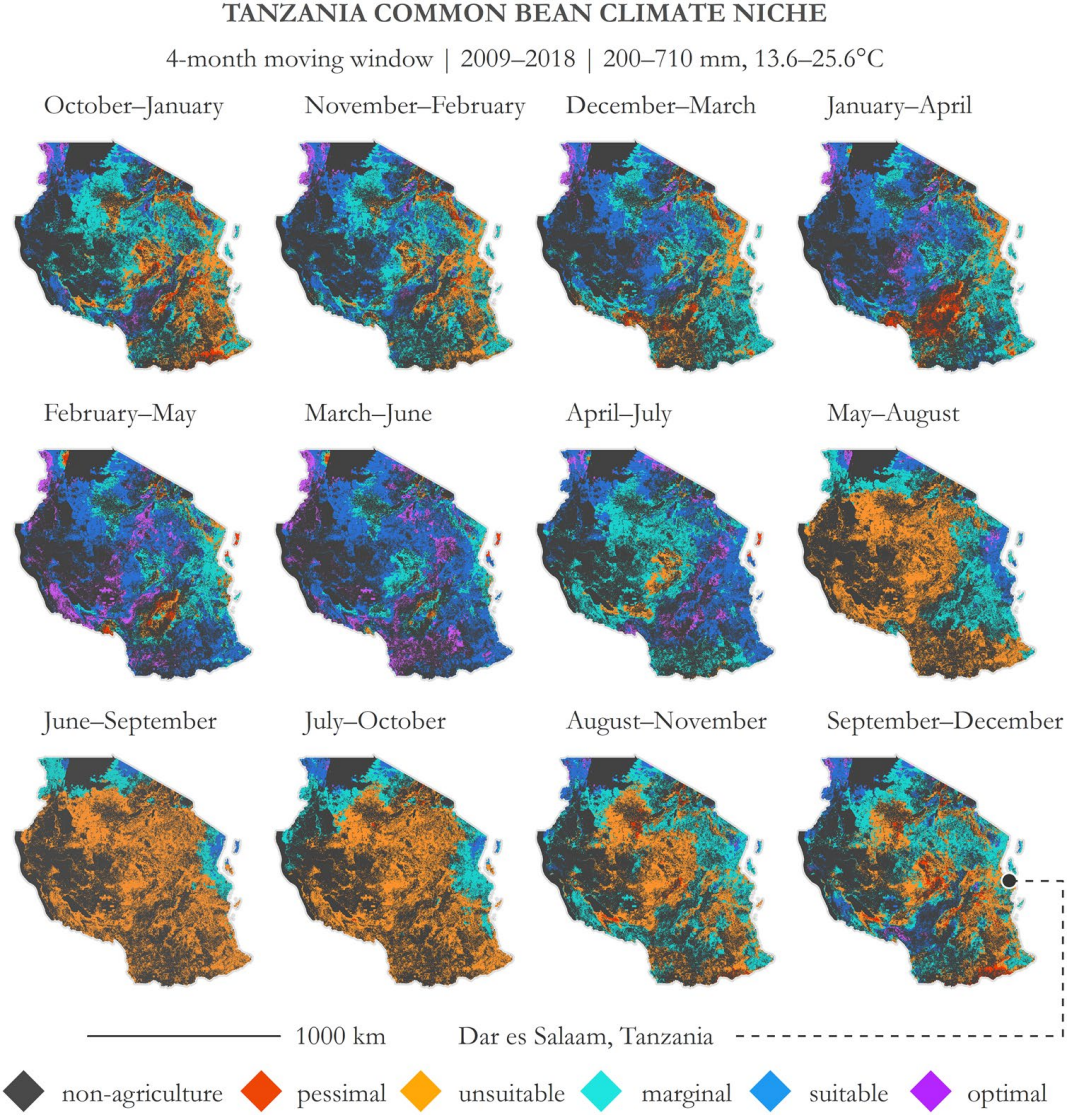
Panel of common bean niche in Tanzania across multiple temporal periods at 4-month intervals. Temporal range: 2009–2018; precipitation range: 200–710 mm; temperature range: 13.6–25.6 °C. Note that there is a slight difference in the temporal periods used for these maps as compared to Figs. 1–4. October–January, November–February, and December–March use 2008–2017, whereas the remaining month ranges use 2009–2018. This decision was made so that the moving interval windows would be sequential. Imagery processed using GEE^38^ and figure prepared using ArcGIS^96^. Data sources: CHIRPS precipitation^67^ and NASA MODIS LST^85^.

We illustrate the temporal capabilities of the application (and management of MTUP) through common bean suitability maps constructed for Tanzania along 4-month moving window intervals (Fig. 5). Suitability category land area proportions are tabulated in Table 2. Using these parameters, January–April (50.4% suitable land area), February–May (66.8% suitable land area), and March–June (79.1% suitable land area) show the greatest potential for common bean cultivation. Conversely, June–September is the least suitable period, with only 3.3% of the land area suitable for common bean cultivation; suitable areas are located primarily along the oceanic coast and near Lake Victoria. This monthly moving window approach may also be used to elucidate areas that are never suitable, as well as areas that are suitable for multiple monthly range periods. In this particular case, 8.7% of agricultural land in Tanzania is never suitable for common bean production; conversely, 91.3% is suitable for at least one of the monthly range periods. Overall, 21.2% of agricultural land in Tanzania is suitable for at least 6 of the monthly growing period windows; 48.3% is suitable for at least 4 of the monthly growing period windows.

**Table 2:** Suitability category proportions on agricultural land corresponding to Fig. 5**.** Subscript S = suitable (optimal and suitable) and subscript N = not suitable (marginal, unsuitable, and pessimal). Each column represents a month range interval labeled with numerical headers (1 = January and 12 = December).

	Land area proportion (%) by moving month range intervals*											
	10–1	11–2	12–3	1–4	2–5	3–6	4–7	5–8	6–9	7–10	8–11	9–12
Optimal	3.3	3.4	3.7	5.7	13.8	14.9	6.2	1.0	< 0.1	0.1	0.5	1.4
Suitable	18.7	29.1	36.6	44.7	53.0	64.2	44.0	9.7	3.3	5.3	6.9	14.4
***Total***_***S***_	*22.0*	*32.5*	*40.3*	*50.4*	*66.8*	*79.1*	*50.2*	*10.7*	*3.3*	*5.4*	*7.4*	*15.8*
Marginal	45.9	44.2	41.6	34.5	26.4	18.9	43.7	31.0	13.5	21.9	43.0	43.2
Unsuitable	28.6	22.0	16.3	11.0	5.2	1.4	5.8	58.3	83.2	72.5	47.9	36.9
Pessimal	3.4	1.3	1.8	4.1	1.6	0.6	0.3	0	< 0.1	0.2	1.6	4.1
***Total***_***N***_	*77.9*	*67.5*	*59.7*	*49.6*	*33.2*	*20.9*	*49.8*	*89.3*	*96.7*	*94.6*	*92.5*	*84.2*

**Figure 6:**
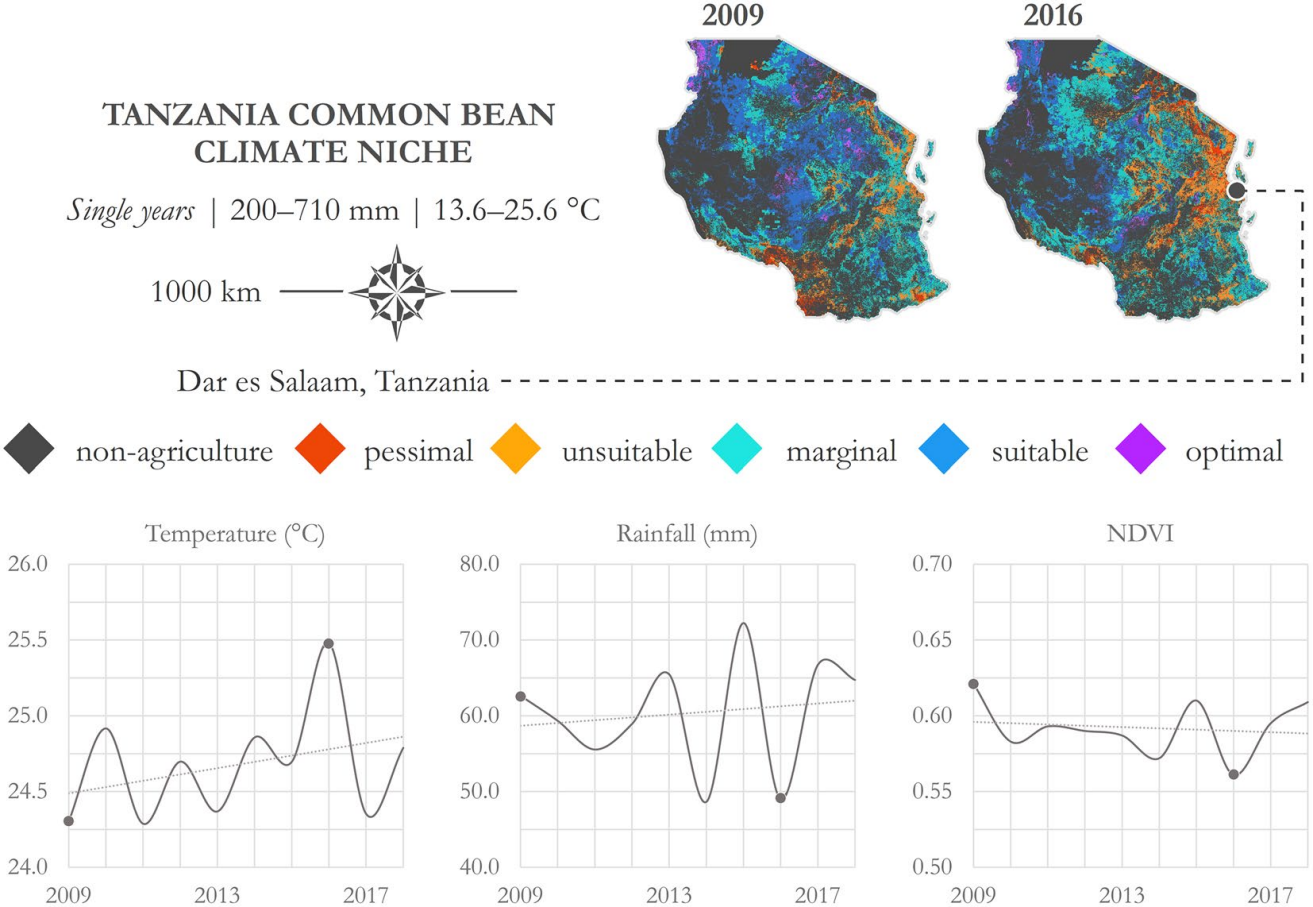
Crop suitability during climate extremes. Growing season months: December–March; temporal units: 2009 and 2016; precipitation range: 200–710 mm; temperature range: 13.6–25.6 °C. Imagery processed using GEE^38^ and figure prepared using ArcGIS^96^. Data sources: CHIRPS precipitation^67^, NASA MODIS LST^85^, and NASA MODIS NDVI^86^.

Hallmarks of pulse crops such as common bean, cowpea, and pigeonpea are unique traits that make them resilient to marginal environments (i.e., stress imposed by periodic droughts, elevated temperatures, and poor soil quality). Thus far in the manuscript, results have been presented that identify crop suitability based on decadal climate characterizations. In this case, climate extremes may be softened; however, one of the novel insights afforded by this application comes from complete control over the temporal units used for aggregation. An example of this flexibility is outlined in Fig. 6, where first a decade of climate data (December–March of 2009–2016) were queried to retrieve time-series charts of temperature, rainfall, and NDVI. These data are presented in the application UI and are also available for download as CSVs. A marked low point in NDVI is observed in 2016 and a high point is observed in 2009. The low point in 2016 corresponds with particularly low rainfall and high temperatures; 2009 corresponds with lower temperatures and a moderate amount of rainfall. Crop suitability was mapped for these two years individually and juxtaposed in Fig. 6. The purpose of the 2016 map in particular is to highlight the potential geographic niche of common bean during a year that experienced climate extremes. It can be seen that more of the country falls into a not suitable status (i.e., marginal, unsuitable, or pessimal) as compared to 2009; however, 27.6% of agricultural land across Tanzania remained suitable for common bean during this particularly marginal production year. In contrast, 45.7% of agricultural land in Tanzania was suitable for common bean production during 2009.

## Discussion and conclusions

In this paper, we introduced a novel tool that addresses the ‘when’ and ‘where’ of crop climate-suitability, to facilitate practitioners’ access to geospatial patterns and trends. Limitations of conventional crop technology scaling has been singled out as a key challenge in achieving sustainable development goals and a solution is offered here for identifying where innovative technologies might be suitably deployed. The application presented demonstrates how practitioners can access geospatial data to generate map products and geovisualizations that investigate crop suitability with agile date and crop phenology parameters. Further, sustainable development practitioners can use this model to elucidate scalability and suitability of future crop varieties that are genetically engineered to tolerate marginal environments. Furthermore, this application manages MTUP by giving complete control to the user to decide how seasons are defined and which years to assess.

Until recently, targeting deployment of natural solutions has been addressed primarily through assessing current cropping patterns, desktop computer-based simulations, or based on anecdotal evidence rather than direct measurements^29^. These practices may be indicative of what grows where, or what theoretically could be grown, but they preclude interactive geovisualizations and spatiotemporal continuity. In contrast, the web-based GIS presented here supports interactive mapping and user parameterization of suitability that can be queried for information, within an internet browser, to help guide decision making and technology development. Our product uses an approach popularized by digital media, whereas until now academic journals have been the dominant platform for communication in geospatial science. This has meant that a large proportion of map-based geographic science has been delivered at the scale of a printable page, at irregular intervals, and often behind paywall, which has severely limited access in the Global South and those without affiliation to research institutions^74^. Even with access to published map products there are issues with communicating fine spatial resolution imagery, as scaling a figure down for print on standard letter-size paper can result in data loss from resampling.

With advancements in cloud-computing and server-side geoprocessing, data can be retrieved and analyzed at unprecedented scales and speeds. One stark advantage here is that with web-based GIS geocommunication tools, stakeholders can pan, zoom, and query information as they need. The biogeographic crop niche approach presented here is an example of the type of evidence-based products that can now be accessed through web-based GIS tools to guide policy and target implementations. The bean cultivar niche products for Africa shown here illustrate the importance of local specificity, along with the ability to query and alter the geovisualization. It shows the utility of these web-based products in supporting stakeholders' interactive ability to query performance for altered crop genetics and climate conditions.

This model considers seasonal rainfall totals and does not yet account for crop water requirements at critical points during the crop growth cycle. Future heuristics of the kind embedded here will benefit from integration of crop system models that tie daily rainfall and temperature to specific crop timing requirements. At present, some of the hurdles in this area include (1) remotely-sensed climate data at the daily time scale not affected by cloud interference, (2) limited global soil information, and (3) cyberinfrastructure requirements to make such an application open access. Research in this domain will progress as interdisciplinary collaborations emerge among remote sensing-specialized geographers and agroecologists partnering with software engineers and user experience designers. Further verification of relationships between crop suitability models and field experimentation will strengthen this application and it should be acknowledged that it is challenging to disentangle climate suitability, as many factors influence yields across geographic regions. Like many crop suitability models, the one presented here relies on the accuracy of the temperature and rainfall estimates used as inputs, as well as accuracy of the field-based and laboratory testing used to measure optimal climate requirements for individual crop varieties.

Spatially continuous soils data that are accurate at local scales is an area of ongoing research and should be prioritized. At present, soil information in the crop niche application presented here is limited to query only, primarily due to the scant database resources available that quantify optimum soil nutrient ranges unique to each crop, as well as accuracy challenges in areas of limited sampling; however, a future iteration of this model will benefit from terrain and soil information to further contextualize environmental suitability across spatial and temporal scales.

### Geographic tools for transdisciplinary agronomy

Sustainable development requires access to scientific knowledge, yet access to, and utilization of, publicly available open data has only begun to be explored and its impact remains contested^75^. An accessible evidence base that can be interactively visualized can provide a critical input to support local food security solutions. Educators and community development actors in particular lack access to such tools, which could be key to timely climate change adaptation and sustainable development^76^. Transdisciplinary teams and community partners require information that can effectively address the highly specific edaphic properties and climate of their locality. Local, regional, and broader scale efforts could all benefit from continuously updatable geospatial data, quantitative metrics, and map products regarding biophysical niche, agricultural productivity, and food security.

In this case example, the country context matters in terms of where an improved bean cultivar with heat tolerance could be introduced. This varies with locality, as can be shown for a range of time and space scenarios using this geographic tool. In some countries, a genetic alteration in common bean heat tolerance makes no discernable difference in the suitability niche. In the hot dry environments of Tanzania, a heat tolerant bean cultivar could substantially expand the crop production niche. In support of climate change adaptation and development goals, earlier research has highlighted that heat tolerance in common bean could expand the niche for this important crop in East Africa, though the impact of this map product was limited due to its static nature and resolution issues typical of journal publications^55^. This lack of accessibility to policy makers and other stakeholders is widespread, as shown by earlier research on the soybean adoption niche in Africa^77^, chickpea adoption and yield gaps in India^78^, and our own earlier research on African pigeonpea and sorghum suitability niches^44^.

Crop system biodiversity has been regarded as a prominent mechanism for enhancing farm resilience to environmental stressors and variable climate^6,58^. Though common bean was the focus here, other legumes and grains can be similarly evaluated. A future implementation of this tool could include multiple crop evaluations to determine where suitability converges (e.g., maize, pigeonpea, and sorghum systems that generate calories and protein for intake, enrich soils via nitrogen fixation and root biomass, and prevent soil nutrient loss from erosion). Of course, there are many factors to consider when proposing new crop introductions or reintegration; however, climate suitability is a fundamental necessity for crop production^24^. In addition, soil properties, economic infrastructure, and farmer preference require attention. In Malawi, for example, pigeonpea uptake varies regionally, owing in part to existing market infrastructure in the southern districts that enable profitability^79^, as well as an established food culture that incorporates pigeonpea in household consumption^80^. For these reasons, effective crop system biodiversity will benefit from interdisciplinary research that engages key stakeholders and includes participatory research with farmers^59^. Furthermore, it is recommended that suitability tools such as the kind supplied here are not used as a sole resource for top-down regulation that could further biodiversity loss^81^.

Successful adoption of new technologies and large-scale agricultural adaptation will benefit from geovisualizations of crop suitability niches. This is often a knowledge gap faced even by holistic approaches where multiple disciplines and stakeholders converge to address food security challenges. We contribute this product generation for biogeographic crop suitability niche as real-time information that can be queried, panned, and simulations run with different climatic parameters. Practitioners and stakeholders in sustainable development can use this as input alongside an understanding of the social dimensions, e.g., labor requirements, market structures, gendered responsibilities, infrastructure, and economy, as all are critical for alleviating food insecurity and designing systems that are sustainable across multiple domains^21^.

## Methods

### Leveraging big data for crop climate-suitability characterization

GEE was the GIS platform selected for this application because it is open access, contains a large repository of global geospatial remote sensing data, and supports the ingestion of new and improved data into existing models at regular temporal intervals. Thus far, GEE has been tapped successfully for a wide range of applications across many disciplines, including water cycle process modeling, global vegetation and agriculture monitoring, and ecological niche mapping for a variety of plant and animal life, some of which are featured on the GEE website (see: https://earthengine.google.com/case_studies/). Recent studies utilizing GEE have also mapped global surface water and evaluated decadal landscape changes^82,83^, and Allen et al.^84^ devised a remote sensing-derived model to map land surface evapotranspiration (*EEFlux*).

All data used in this application are hosted by GEE (Table 3). Data products used (summarized in detail in following sections) included UCSB Climate Hazards Group precipitation (CHIRPS)^67^, NASA MODIS temperature (MOD11A2)^85^, NASA MODIS vegetation indices (MOD13Q1)^86^, NASA MODIS land-cover type (MCD12Q1)^87, 88^, NASA/USGS cropland extent (GFSAD)^89^, ESA land-cover type (GlobCover)^90^, OpenGeoHub/LandGIS soil properties^91^, and NASA/NGA/DLR/DET/ASI elevation (SRTM)^92^. OpenGeoHub/LandGIS soil properties include organic carbon, bulk density, pH in H2O, soil water content, sand/silt/clay fraction, and biome/taxonomy groupings.

**Table 3:** List of data products and acronyms. ASI (Agenzia Spaziale Italiana—Italian Space Agency), CHIRPS (Climate Hazards Group InfraRed Precipitation with Station data), DLR (Deutsches Zentrum für Luft- und Raumfahrt—German Aerospace Center), ESA (European Space Agency), GFSAD (Global Food-Support Analysis Data), MODIS (Moderate Resolution Imaging Spectroradiometer), NASA (National Aeronautics and Space Administration), NGA (National Geospatial Intelligence Agency), SRTM (Shuttle Radar Topography Mission), UCLouvain (Université catholique de Louvain), UCSB (University of California, Santa Barbara), USGS (United States Geological Survey). *OpenGeoHub/LandGIS soil properties include organic carbon, bulk density, pH in H2O, soil water content, sand/silt/clay fraction, and biome/taxonomy groupings—current products utilize data ranging from January 1, 1950 to January 1, 2018.

Data	Source	Product	Temporal range	Temporal resolution	Spatial resolution
Rainfall	UCSB Climate Hazards Group	CHIRPS Pentad	1981–01-01 to present	5-day	~ 5.5-km
Temperature	NASA MODIS	MOD11A2 v006	2000–02-18 to present	8-day	1-km
NDVI	NASA MODIS	MOD13Q1 v006	2000–02-18 to present	16-day	250-m
Agricultural land-use	NASA MODIS	MCD12Q1 v051 MCD12Q1 v006	2000–2013 2000–2018	Annual	500-m
	ESA/UCLouvain	GlobCover 2009	2009	Annual	300-m
	NASA/USGS	GFSAD1000	2000	–	1-km
Soil properties*	OpenGeoHub/LandGIS	OpenLandMap	*	–	250-m
Elevation and slope	NASA/NGA/ DLR/DET/ASI	SRTM	2000	–	30-m

**Figure 7:**
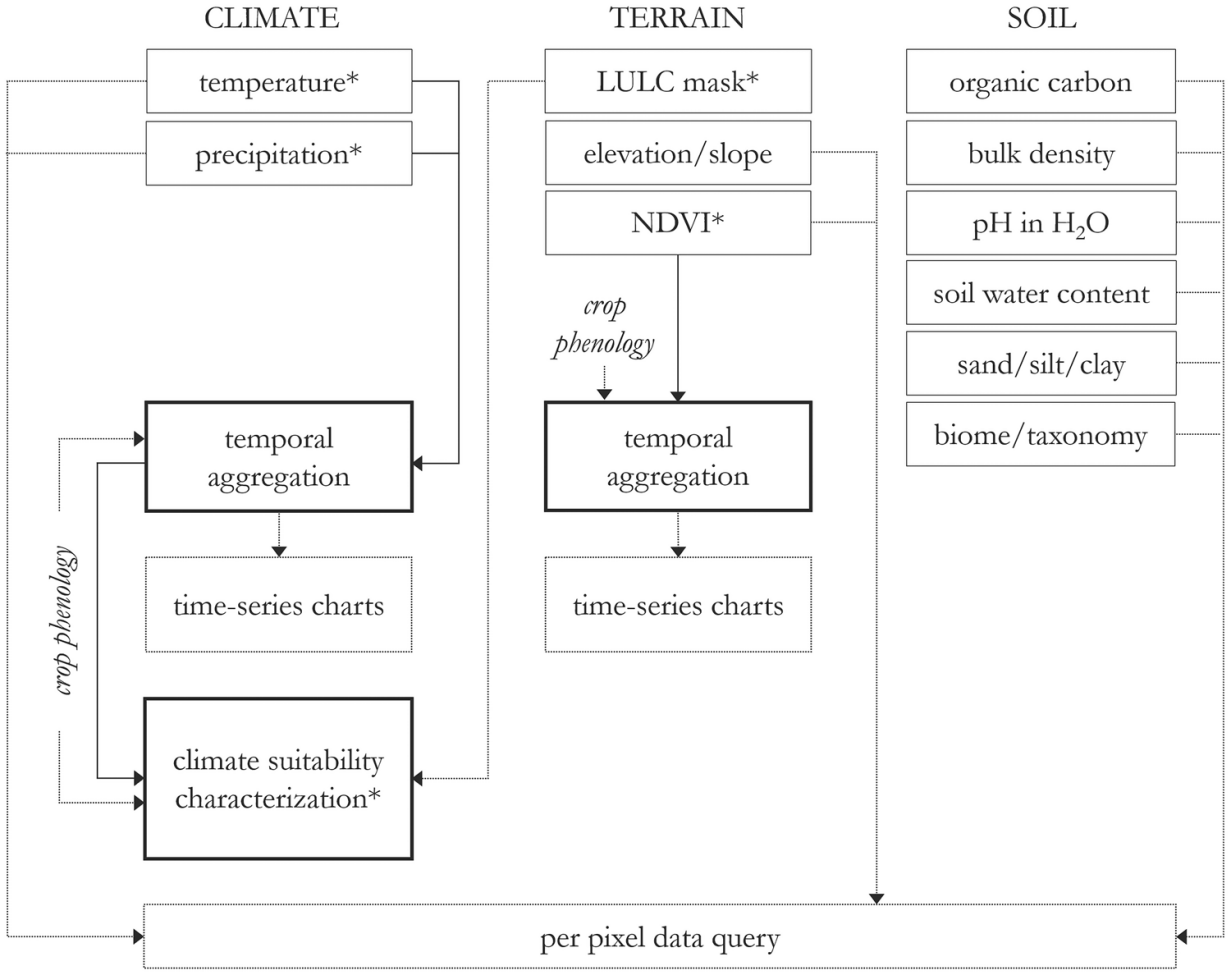
Workflow diagram. Asterisk indicates data products that are included as map outputs.

The workflow involves (1) acquiring temperature, precipitation, and NDVI for the date range and season range selected, (2) parsing temperature and rainfall into suitability classifications based on user-selected crop phenology parameters, (3) temperature, precipitation, and NDVI time-series charts at the pixel and regional level, and (4) optional agricultural land masking and querying of each data product at the pixel level (Fig. 7).

### Data products: temperature, precipitation, soil, terrain, and land-use/land-cover

The precipitation data product used here is the Climate Hazards Group InfraRed Precipitation with Station data version 2.0 (CHIRPS) from the University of California Santa Barbara Climate Hazards Group (UCSB-CHG). CHIRPS is a quasi-global precipitation model that pairs 0.05-degree spatial resolution remote sensing imagery with in situ weather station data^67^. The latitudinal extent of CHIRPS is between 50°N and 50°S, and is supplied through GEE at a spatial resolution of approximately 5.5 km. CHIRPS is delivered at two temporal resolutions through GEE—daily and pentad; the pentad temporal resolution was selected here to minimize processing times for global-scale analysis; however, a daily temporal resolution can be selected with minimal modification to the code. Seasonal total rainfall is calculated using input month range selections, as well as the average seasonal rainfall across the complete temporal range.

Temperature estimates were retrieved from the MODIS Terra Land Surface Temperature (LST) product (MOD11A2 V006)^85^. An 8-day temporal resolution was selected here to minimize processing time for global-scale analysis. The temporal resolution of MODIS LST can be changed with minimal modifications to the code; however, there is a substantial amount of cloud cover over some regions during their respective growing seasons in the daily product, making it potentially only marginally more accurate. Day and night temperatures are averaged over the growing season date range selected by the user. Average seasonal rainfall amounts and average seasonal temperature across the selected date ranges and temporal period are mapped as outputs in the application. NDVI data were retrieved from the MODIS Terra Vegetation Indices product (MOD13Q1 V006)^86^.

An area of advancement in modern precision agriculture is the refinement of soil sensing at local scales^93^. Agricultural land suitability has conventionally relied on regional-scale soil classifications; however, cost effective soil data collection devices have enabled farmers and researchers to measure soil characteristics such as salinity, organic carbon, moisture, and texture at the individual plant scale. Moreover, collective efforts (including citizen science), such as OpenGeoHub LandGIS (https://opengeohub.org/)^91^ and LandPKS^94^, have designed platforms that enable users to contribute detailed field soil sampling. The recently available OpenLandMap soils products are unique in that machine learning algorithms enable regular improvement as more field samples are collected. The OpenLandMap processes and accuracy metrics are detailed by Hengl and MacMillan^91^, which is available online at https://soilmapper.org/. Soils data retrievable in this application from OpenGeoHub LandGIS are organic carbon, bulk density, pH in H_2_O, soil water content, sand/silt/clay fractions, and biome/taxonomy categories. Elevation and slope data are retrieved from the USGS Shuttle Radar Topography Mission (SRTM)^92^.

Agricultural land is delineated here using GlobCover 2009 (S1)^90^, MODIS Land Cover Type (MCD12Q1 V051 (S2) and V006 (S3))^87,88^, and the GFSAD1000 Cropland Extent (S4)^89^ products; S1–4 are referenced as subscripts later in this paragraph. All four land-cover products were combined to minimize errors of omission. For MCD12Q1 time-series LULC, the mode was used. If the land is classified as agriculture (or partially agriculture) in any system, it is demarcated as agricultural land. This approach was selected to maximize the amount of agricultural land identified, rather than excluding or underestimating agricultural lands. One limitation of this approach is that agricultural lands may be overestimated and subject to errors of commission in some regions; however, using any single classification system is also affected by the same under or overestimation problems. The procedure for this classification is as follows, where 0 is non-agriculture and 1 is agriculture and the subscript is the classification system: [0, 1]_S1_ + [0, 1]_S2_ + [0, 1]_S3_ + [0, 1]_S4_ = [0, 1, 2, 3, 4], then [0, 1, 2, 3, 4] → [0, 1, 1, 1, 1] → [0, 1]. The agricultural land-cover dataset produced is used as an optional mask in the application. Note that MCD12Q1 V051 is now considered superseded by MCD12Q1 V006 and was removed from the GEE catalog during the revision stage of this manuscript. The application continues to function using only V006 of the MCD12Q1 data product and the model is conceptually unchanged.

### Common bean phenological requirements

Common bean is one of the most widely cultivated legume crops across the globe and is noted for its biological fixation of nitrogen^53^. It has been genetically modified as an early-maturing crop that is resilient to abiotic stressors such as drought, high altitudes, disease, and nitrogen deficiency^54^. Parameterization for the fundamental climate niche of common bean used here was gathered from Beebe et al.^72^, where suitable temperature and precipitation ranges during a growing season are 13.6–25.6 °C and 200–710 mm, respectively. A hypothetical scenario was also tested for Tanzania to elucidate where a common bean variety genetically engineered to withstand hotter temperatures could feasibly grow. The hypothetical scenario extends the maximum temperature to 27.6 °C. Common bean was used as a case study here; however, Pironon et al.^95^ have shown adaptive strategies exist for integrating many legume and grain crops (e.g., pigeonpea and sorghum) in Sub-Saharan African agriculture.

A growing season of December–March was used here, which coincides with the rainfall and production trends in Southeastern Africa^68,69^—a decade of crop production seasons was used to typify the climate. Data used range from 2009-12-01 to 2019-03-31 (April–November months excluded). To clarify, the following seasons were used: [2009-12-01 to 2010-03-31], [2010-12-01 to 2011-03-31], [2011-12-01 to 2012-03-31], [2012-12-01 to 2013-03-31], [2013-12-01 to 2014-03-31], [2014-12-01 to 2015-03-31], [2015-12-01 to 2016-03-31], [2016-12-01 to 2017-03-31], [2017-12-01 to 2018-03-31], and [2018-12-01 to 2019-03-31]. Climate data are first aggregated by season, then aggregated across the temporal range before parsing suitability classifications. The same protocol and parameters that were used to map the suitable area in Tanzania were also used to map the suitable area across all of Southeastern Africa; countries include South Africa, Tanzania, Mozambique, Zambia, Madagascar, Botswana, Kenya, Zimbabwe, Uganda, Malawi, Lesotho, Burundi, Rwanda, and Swaziland.

### Parsing suitability classifications

**Figure 8:**
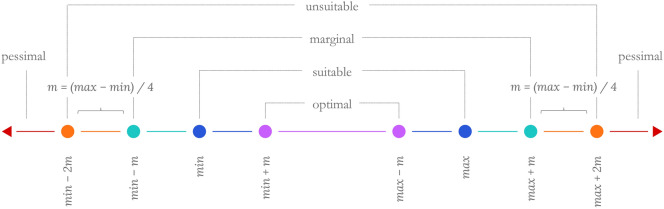
Diagram depicting generalizable method for parsing suitability classifications.

There are two overarching suitability classifications—suitable or not suitable. A range-based approach was used to parse crop suitability into five categories: optimal, suitable, marginal, unsuitable, and pessimal^44^; the binary suitable and not suitable categories are also supplied in the application. Optimal and suitable sub-categories fall under suitable (S) and the marginal, unsuitable, and pessimal categories fall under not suitable (N). The inputs entered by the user are the suitable min and max represented by the blue dots in Fig. 8. To generate the five classes, a range modifier value (m) was computed by dividing the range of the input min and max by 4. The modifier is used to extrapolate the range outward toward marginal, unsuitable, and pessimal, and interpolate inward toward optimal^44^. While suitability thresholds will vary by crop, this method offers a generalizable approach to visualizing crop suitability beyond binary categories. Suitability ranges are defined by the following formulae and represented graphically in Fig. 8:

$$\begin{aligned} & {\text{m }} = \, \left( {{\max}{-}{\min}} \right)/{4} \\ & {\text{optimal }} = \, \{ {\text{x}} \in {\mathbb{R}}|{\min} + {\text{ m }} < {\text{ x }} < {\max}{-}{\text{ m}}\} \\ & {\text{suitable }} = \, \{ {\text{x}} \in {\mathbb{R}}|{\min} < {\text{ x }} < {\min} + {\text{ m OR max }}{-}{\text{ m}} < {\text{ x }} < {\max}\} \\ & {\text{marginal }} = \, \{ {\text{x}} \in {\mathbb{R}}|{\min}{-}{\text{ m }} < {\text{ x }} < {\text{ min OR max }} < {\text{ x }} < {\max} + {\text{ m}}\} \\ & {\text{unsuitable }} = \, \{ {\text{x}} \in {\mathbb{R}}|{\min}{-}{\text{ 2m }} < {\text{ x }} < {\min}{-}{\text{ m OR max }} + {\text{ m }} < {\text{ x }} < {\max} + {\text{ 2m}}\} \\ & {\text{pessimal }} = \, \{ {\text{x}} \in {\mathbb{R}}|{\text{ x }} < {\min}{-}{\text{ 2m OR x }} > {\max} + {\text{ 2m}}\} \\ \end{aligned}$$

Suitability characterizations were first generated for temperature and precipitation individually before being combined. After temperature and precipitation suitability characterizations are produced, they are compared using a minimum argument so that the combined suitability classification is the minimum of either product: e.g., min([2, 1, 4, 3, 5]_T_, [5, 1, 4, 2, 5]_P_) = [2, 1, 4, 2, 5]_C_, where T = temperature, P = precipitation, C = combined, 1 = pessimal, 2 = unsuitable, 3 = marginal, 4 = suitable, and 5 = optimal.

## Data Availability

Code associated with this manuscript can be accessed via Harvard Dataverse at https://doi.org/10.7910/DVN/UFC6B5. Application development and data sharing was made possible by partnership with the Feed the Future Innovation Lab for Collaborative Research on Sustainable Intensification (https://www.k-state.edu/siil/); grant details are listed in the acknowledgements.
